# Central node dissection in papillary thyroid carcinoma in the era of near-infrared fluorescence

**DOI:** 10.3389/fendo.2023.1110489

**Published:** 2023-04-14

**Authors:** Paulina Kuczma, Marco Stefano Demarchi, Sophie Leboulleux, Christophe Trésallet, Maria Mavromati, Reza Djafarrian, Andrea Mabilia, Frédéric Triponez

**Affiliations:** ^1^ Department of Thoracic and Endocrine Surgery, University Hospitals and Faculty of Medicine of Geneva, Geneva, Switzerland; ^2^ Department of Endocrinology, University Hospitals and Faculty of Medicine of Geneva, Geneva, Switzerland; ^3^ Assistance Publique–Hôpitaux de Paris, Department of Digestive, Bariatric and Endocrine Surgery, Bobigny Avicenne Hospital, Sorbonne Paris Nord University, Bobigny, France

**Keywords:** lymph node dissection, papillary thyroid carcinoma/surgery, papillary thyroid cancer, near-infrared fluorescence (NIR) imaging, ICG angiography, central lymph node dissection (CLND), prophylactic central lymph node dissection

## Abstract

The most common site of lymph node metastases in papillary thyroid carcinoma is the central compartment of the neck (level VI). In many patients, nodal metastases in this area are not clinically apparent, neither on preoperative imaging nor during surgery. Prophylactic surgical clearance of the level VI in the absence of clinically suspicious lymph nodes (cN0) is still under debate. It has been suggested to reduce local recurrence and improve disease-specific survival. Moreover, it helps to accurately diagnose the lymph node involvement and provides important staging information useful for tailoring of the radioactive iodine regimen and estimating the risk of recurrence. Yet, many studies have shown no benefit to the long-term outcome. Arguments against the prophylactic central lymph node dissection (CLND) cite minimal oncologic benefit and concomitant higher operative morbidity, with hypoparathyroidism being the most common complication. Recently, near-infrared fluorescence imaging has emerged as a novel tool to identify and preserve parathyroid glands during thyroid surgery. We provide an overview of the current scientific landscape of fluorescence imaging in thyroid surgery, of the controversies around the prophylactic CLND, and of fluorescence imaging applications in CLND. To date, only three studies evaluated fluorescence imaging in patients undergoing thyroidectomy and prophylactic or therapeutic CLND for thyroid cancer. The results suggest that fluorescence imaging has the potential to minimise the risk of hypoparathyroidism associated with CLND, while allowing to exploit all its potential benefits. With further development, fluorescence imaging techniques might shift the paradigm to recommend more frequently prophylactic CLND.

## Introduction

Thyroid cancer is the most common endocrine malignancy worldwide and the 9^th^ most common malignancy in Europe and Northern America (1). Incidence varies across the world, with the highest reported in South Korea (111.3/100 000), where thyroid cancer is the most common cancer in women, due to excessive screening. In most countries, the incidence of thyroid cancer has been clearly on the rise in the last decades. In the United States, the incidence tripled between 1975 and 2009, and the steepest, about 10-fold increase caused by overdiagnosis was observed in South Korea (2). This increase is attributed almost exclusively to papillary thyroid cancer (PTC), which accounts for from 85% to over 95% of thyroid cancer diagnoses (3, 4). In the vast majority of cases, however, the prognosis remains excellent and the mortality is low.

Surgery constitutes the most commonly used curative treatment for PTC. Depending on the preoperative staging, lobectomy or total thyroidectomy is recommended (5). Lymph node metastases in PTC are very common and present in the majority of patients at the moment of diagnosis (6). In the case of preoperatively or intraoperatively discovered lymph node metastases, the recommendation concerning the lymph nodes is clear: therapeutic compartment-oriented dissection (5). Prophylactic central lymph node dissection (CLND), however, is still debated, mainly due to the fact that it increases the frequency of postoperative hypoparathyroidism.

Fluorescence imaging, a relatively new tool in endocrine surgery, facilitates the identification and preservation of parathyroid glands and can contribute to lower frequency of postoperative hypoparathyroidism. It can therefore be of great use to minimise the risks associated with CLND, while allowing to take advantage of all its potential benefits.

The aim of this review is to synthesise the current scientific literature on the use of fluorescence imaging in thyroidectomy with lymph node dissection, and to examine the arguments for and against prophylactic CLND in the light of reported outcomes of this imaging technique.

## Lymph node metastases in PTC and their impact on patient outcomes

The frequency of lymph node metastases in PTC ranges from 44.5% to more than 60% (6–8) and tends to decrease with age (7). Also microscopic lymph node metastases in papillary thyroid microcarcinoma are common: they were present in 40% to 64% of patients who underwent central neck dissection (8). Metastatic lateral lymph nodes are found during prophylactic neck dissection in up to 44% of patients with papillary thyroid microcarcinoma (9). Despite the extremely frequent nodal involvement, occurring even at early disease stages, the prognosis is excellent, with 10-year disease-specific survival reaching 98.6% (10).

The impact of lymph node metastasis on patient outcome has been extensively studied, including in large epidemiological studies. Most studies report that the presence of metastatic lymph nodes is associated with lower survival and/or higher disease recurrence (11–16), while others report similar outcomes in patients with and without lymph node metastases (17). However, data from large epidemiological studies seem to outweigh the doubts, suggesting that metastases are associated with worse patient outcomes (11–13, 15). Besides, most studies do not differentiate between preoperatively diagnosed, pathologically-diagnosed macroscopic and microscopic lymph node metastases.

## Prophylactic central neck dissection – definition and debate

Therapeutic lymph node dissection is systematically performed for clinically apparent metastases (cN1) identified either preoperatively by imaging or palpation, or intraoperatively by the surgeon. Dissection of clinically uninvolved lymph nodes (cN0) is called prophylactic neck dissection. Compartment-oriented therapeutic neck dissection for the central and lateral compartments of the neck is currently not subject to debate, and is recommended by most guidelines and societies, including the 2015 ATA guidelines (5). Prophylactic lateral neck dissection is not recommended, because the lateral neck constitutes a separate compartment, which remains untouched during the initial thyroidectomy, so it can be dissected at a later stage without any significant additional risk. On the other hand, prophylactic CLND has been the subject of extensive scientific discussion which has so far led to no clear consensus. According to the ATA 2015 guidelines, prophylactic CLND should be considered for patients with advanced primary tumours (T3 and T4), or if the information obtained will be used to plan further therapy (5).

The rationale for prophylactic CLND is the high rate of occult nodal metastases in PTC (up to 40%-60%) (6–8) and the lack of a reliable method to detect them preoperatively or intraoperatively. Preoperative ultrasonography detects only up to 20% of involved lymph nodes, and its sensitivity is limited, particularly in the central compartment, due to the overlying thyroid gland. Ito et al. calculated that preoperative ultrasonography detects central lymph node metastases with 99% specificity, but only just over 10% sensitivity (18). Prophylactic CLND provides precise staging information, which can guide the decision to administer radioactive iodine postoperatively. The size, number and ratio of metastatic lymph nodes are important factors in assessing the risk of recurrence (19). This information about affected lymph nodes in the central compartment can be accurately obtained only through CLND. Bonnet et al. found that lymph node status affected the decision on postoperative radioactive iodine treatment in 30.5% of patients with PTC <2 cm (20). The value of more precise risk estimation lies not only in identifying patients who are at higher risk of recurrence and would benefit from radioactive iodine treatment, but also in reliably selecting those for whom radioactive iodine therapy can be omitted, avoiding overtreatment. In the study by Bonnet et al., radioactive iodine treatment could have been avoided in 42% of patients when the pN0 status and other low-risk features were confirmed. At the time of the study, however, radioactive iodine was given to all patients with lymph node metastases who had intermediate- or high-risk cancers as defined in the 2009 ATA and 2006 European recommendations (21, 22). Conversely, in low risk thyroid cancer patients, an ipsilateral prophylactic CLND upstages 9% of the patients to intermediate risk (23). However, in a recent randomized controlled clinical trial by Leboulleux et al., follow-up without radioactive iodine therapy was non-inferior to follow-up with radioactive iodine ablation in terms of recurrence outcomes in patients with low-risk differentiated thyroid cancer and Nx/N0 status (24).

More precise diagnosis of the lymph node status provides accurate indications not only for radioactive iodine treatment, but also for adequate initial staging and follow-up. The initial pN0 status in otherwise low-risk tumours might obviate the need for repetitive exams and associated high healthcare costs, while, the pN1 status might lead to a more careful follow-up.

Many retrospective studies have investigated the impact of CLND on patient outcomes (i.e. recurrence and survival), but the results are conflicting. Contrary to what was previously claimed (25), prospective trials on the effects of CNLD are feasible. An ongoing randomized clinical trial (ESTIMABL 3) studies prophylactic CLND for thyroid cancer with the frequency of excellent response after total thyroidectomy with or without CNLD and surgical morbidity as major endpoints (NCT03570021). After analysing thirty retrospective studies and five meta-analyses, the European Society of Endocrine Surgeons issued a consensus report stating a lower risk of recurrence after prophylactic CLND and recommending that CLND should be performed by surgeons with sufficient experience (26). A recent meta-analysis of 17 studies and 4437 patients who underwent thyroidectomy with and without prophylactic CLND for PTC found a significantly higher risk of recurrence in the latter group (27). Another meta-analysis of 13 studies and 3331 patients found that those treated with thyroidectomy and prophylactic CLND for PTC had the risk of recurrence reduced by 35% (28). A number of groups showed significantly lower recurrence in the central compartment of the neck after CLND (26, 29–31), while others observed no impact of CLND on local recurrence (32–36). Concerning survival, most studies consistently report that the impact of prophylactic CLND on disease-specific survival is limited or negligible (29, 37, 38).

## Postoperative hypoparathyroidism

A commonly mentioned argument against CLND is the higher morbidity burden, particularly of hypoparathyroidism. In a meta-analysis of 13 studies, Lang et al. showed that hypocalcaemia was the main postsurgical morbidity in patients undergoing thyroidectomy with prophylactic CLND for PTC, and that when it was excluded, the overall morbidity was similar to patients treated with thyroidectomy alone (28). A number of studies, including the consensus report of the European Society of Endocrine Surgeons, reported no difference in frequency of permanent hypoparathyroidism in patients who underwent thyroidectomy with and without CLND (26, 29, 31, 39, 40).

Other studies found a significantly higher rate of permanent postoperative hypoparathyroidism in patients undergoing thyroidectomy with CLND (35, 38, 41). A recent study using Scandinavian registry data analysed over 700 patients who underwent thyroidectomy with or without CLND for pT1-T3 PTC and found that permanent hypoparathyroidism was significantly higher in the CLND group (6.6 vs 2.3%) (41).

Permanent postoperative hypoparathyroidism has lifelong consequences: higher risk of cardiac and renal morbidities, renal stones, and mental health problems, as well as frequent paraesthesia, basal ganglia calcifications and impaired cognitive function (42, 43). Recent studies showed that permanent postoperative hypoparathyroidism can even be associated with an increased risk of renal insufficiency, any type of malignancy, cardiovascular events and higher mortality rates (44, 45). Moreover, the management of postoperative hypoparathyroidism increases considerably the financial burden on healthcare systems (46).

## Fluorescence imaging

Fluorescence occurs when a substance excited with light of a particular wavelength emits light of a longer wavelength and lower energy. Fluorescence imaging takes advantage of this property by illuminating tissues with a filtered light of a specific wavelength. The target tissue absorbs the light and then emits light of longer wavelength, which is captured and processed. The substance responsible for fluorescence is called “fluorophore” and can be extrinsic, like contrast agents (indocyanine green (ICG) is the most used contrast agent in surgery), or intrinsic, like melanin or porphyrin, which are occurring in tissues naturally.

## Near-infrared autofluorescence of the parathyroid glands

Paras et al. first described the clinical application of near-infrared autofluorescence (NIRAF) in the detection of parathyroid glands (47). When excited with infrared light of 785 nm wavelength, the parathyroid glands emit light of 820 nm wavelength. These findings were subsequently validated in larger patient cohorts, in benign and malignant thyroid disease, as well as in diverse parathyroid pathologies, all consistently showing stronger autofluorescence of healthy and hyperfunctioning parathyroid glands compared to all the other neck tissues, including the thyroid (48–50). Of note, the fluorophore responsible for NIRAF in the parathyroid glands is currently unknown.

A number of studies have shown that mapping of the parathyroid glands position with NIRAF imaging at various stages of thyroidectomy leads to earlier and more frequent identification of the glands, lowers the rate of inadvertent resection, and allows to salvage more glands from the resected specimen (51–53).

Whether intraoperative detection of NIRAF lowers postoperative hypoparathyroidism and hypocalcemia has also been a subject of numerous studies. Most studies, including several randomized controlled trials, consistently show lower frequencies of postoperative transient hypoparathyroidism (54–57). However, some recent randomized controlled trials found no significant difference in postoperative calcium and parathormone concentrations in NIRAF and control groups (58, 59). No reduction in the frequency of permanent hypoparathyroidism has been observed so far (54–56).

The autofluorescence signal of the parathyroid glands remains unaffected by blood perfusion: in order to assess gland vascularity and viability, other methods, in particular ICG angiography, are necessary.

## Contrast-enhanced near-infrared fluorescence

Unlike autofluorescence imaging, contrast-enhanced fluorescence imaging does not exploit the intrinsic properties of the tissue. The technique requires administration of an extrinsic contrast agent – a fluorescent dye. ICG is a fluorescent dye widely used in intraoperative imaging since the 1950s. It has an excellent safety profile, with very few reported adverse reactions (< 1 per 200 000). ICG quickly binds to plasma proteins and remains almost entirely confined to the vascular system. It is taken up by the hepatocytes and excreted into the bile. ICG has a very fast washout and metabolism, so repeated administrations during a single intervention are possible.

ICG angiography can be used at the beginning of the dissection, to identify and preserve well-vascularized parathyroid glands, or after the dissection, to predict postoperative outcomes. Used at the beginning of the surgery, ICG angiography can aid identification of the parathyroid glands’ vessels, and therefore help preserve the parathyroid glands and the vessels during thyroid dissection. Other studies have shown that intraoperative ICG angiography can be used to accurately evaluate parathyroid vascularization and thus gland function after thyroid resection (60–62). Vidal Fortuny et al. conducted a randomized clinical trial which demonstrated that in the case of at least one well-perfused parathyroid glad on ICG angiography, no patient had hypoparathyroidism after total thyroidectomy and that there was no need for blood testing and calcium/vitamin D supplementation (61). This technique can also be helpful in deciding whether a parathyroid gland should be left *in situ* or autotransplanted, depending on its viability.

## Current research on fluorescence imaging in CLND for thyroid cancer

Reports on the use of fluorescence imaging in CLND are scarce. To date, only three studies, all performed in Asia, evaluated the use of NIRAF and/or ICG angiography in thyroidectomy with CLND for thyroid cancer (63–65). All three groups included patients undergoing thyroidectomy with unilateral or bilateral, prophylactic and therapeutic CLND. **Table 1** summarizes basic information about the three studies.

**Table 1:** Basic information about the studies on fluorescence imaging in CLND for thyroid cancer.

Study	Number of patients	Age	Study design	Indication for surgery
**Ouyang et al.,** (62)	81 34 - study group 47 - control group	Study group: 34.4 ± 8.6 Control group: 35.5 ± 9.4	Retrospective cohort study	PTC
**Kim et al.,** (64)	542 261 –study group 281 - controls	Study group: 51.3 ± 12.4 Control group: 52.8 ± 10.9	Retrospective cohort study with historical control group	All thyroid cancer types
**Yin et al.,** (63)	180 90 - study group 90 - control group	Study group: 42.1 ± 9.5 Control group: 42.7 ± 9.1	Randomized controlled trial	PTC

PTC, papillary thyroid carcinoma.

Ouyang et al. evaluated the impact of ICG angiography on the identification and preservation of the parathyroid glands, as well as on the postoperative parathyroid hormone and calcium concentrations in patients undergoing bilateral axillo-breast approach (BABA) robotic thyroidectomy with unilateral and bilateral CLND (both therapeutic and prophylactic) for PTC (63). ICG angiography was used twice, to identify the parathyroid glands and their blood vessels to guide dissection, and to assess parathyroid perfusion after the removal of the specimen (**Table 2**).

**Table 2:** Type of surgery, lymph node dissection, and fluorescence imaging modality used in the reviewed studies.

Study	Surgery	Lymph node dissection	Fluorescence imaging technique	Modalities
**Ouyang et al.,** (62)	BABA robotic thyroidectomy with lymph node dissection	Central, unilateral and bilateral	ICG	(I) before initial dissection to identify parathyroid glands; (II) during dissection to identify and preserve the vascular pedicle; (III) after dissection to evaluate gland perfusion.
**Kim et al.,** (64)	Thyroidectomy with lymph node dissection	Central and lateral, unilateral and bilateral	NIRAF	(I) before initial dissection; (II) after limited dissection, if no glands were found initially; (III) on the specimen.
**Yin et al.,** (63)	Thyroidectomy with lymph node dissection	Central, unilateral and bilateral	NIRAF and ICG	(I) NIRAF imaging before dissection, to identify parathyroid glands; (II) NIRAF imaging of the specimen; (III) ICG angiography to assess gland perfusion after thyroid removal.

BABA, bilat ral axillo-breast approach; ICG, indocyanine green; NIRAF, near-infrared fluorescence.

Kim et al. investigated the impact of NIRAF imaging on postoperative calcium and parathyroid hormone concentrations in a large cohort of patients undergoing thyroidectomy and CLND (unilateral and bilateral) for thyroid cancer (65). The researchers proposed the concept of “parathyroid gland mapping”, that is localisation of the glands through NIRAF imaging at early stages of the dissection, when no glands are yet visible to the naked eye. If the parathyroid glands were not visible at that stage, NIRAF imaging was used a second time, after limited dissection was performed, and the glands previously covered by tissue were exposed. NIRAF imaging was also performed on the specimen, to search for inadvertently resected parathyroid glands (**Table 2**).

Yin et al. conducted a randomized controlled trial of NIRAF imaging combined with ICG angiography in CLND (64). NIRAF imaging was used in the initial stages of the dissection to identify the parathyroid glands and again, after the removal of the specimen. ICG angiography was used at the end of the surgery, to evaluate blood perfusion of the glands and select those qualifying for autotransplantation (**Table 2**).

Ouang et al. showed that the mean number of identified and *in situ* preserved parathyroid glands was higher in the ICG group (3.74 vs 3.15, *p* < 0.001; and 3.12 vs 2.74, *p* = 0.007, respectively). Patient who underwent ICG angiography were also more likely to have all four parathyroid glands identified and preserved. Yin et al. identified and preserved *in situ* more parathyroid glands in the NIRAF than in the control group (3.6 vs 3.2, *p* < 0.001; and 1.3 vs 1.0, *p* < 0.001, respectively). The number of inadvertently resected and salvaged glands were not reported.

Kim et al. found that the rate of inadvertently resected parathyroid glands in the specimen was 6.9% in the NIRAF group and 12.8% in the control group (*p* = 0.021), but observed no differences in the number of identified parathyroid glands.

The reported impact of the introduction of fluorescence imaging protocols on the frequency of postoperative hypoparathyroidism is summarised in **Table 3**.

**Table 3:** Rates of transient and permanent hypoparathyroidism in the reviewed studies.

Study	Transient hypoparathyroidism	Permanent hypoparathyroidism
**Ouyang et al.,** (62)	Study group: 20.6% Control group: 23.4% *p* = 0.764;	None
**Kim et al.,** (64)	Study group: 33.7% Control group: 46.6% *p* = 0.002	Study group: 4.2% Control group: 4.6% *p* = 0.816
**Yin et al.,** (63)	Study group: 27.8% Control group: 43.3% *p* = 0.029	None

## Discussion

The results suggest that fluorescence imaging may reduce temporary hypoparathyroidism in patients undergoing thyroidectomy with CLND. This could happen through several mechanisms: better identification of the parathyroid glands and their vascular pedicles, lower rates of inadvertent parathyroid resection, and facilitated decision-making on parathyroid glands autotransplantation.

Two of the reviewed studies showed that fluorescence imaging helped lower the frequency of hypoparathyroidism. Ouyang et al. observed no statistical difference, possibly due to the small sample size (63). A relatively high initial frequency of temporary hyperparathyroidism (46.6% in the control group) reported by Kim et al. (65) decreased significantly to 33.7% in the NIRAF group, which is similar to the median incidence of transient hypoparathyroidism after conventional thyroidectomy reported in a recent meta-analysis (up to 38%) (66). Ouyang et al. and Yin et al. observed no cases of permanent hypoparathyroidism (63, 64). In the study by Kim et al., the rate of permanent hypoparathyroidism was 4.2% in the NIRAF group, and was not significantly different from the control group’s 4.6% (65). However, certain significant differences between the two groups were identified: larger tumour size, and higher frequency of lymph node metastases in the central and lateral compartments in the NIRAF group. The reported frequency of permanent postoperative hypoparathyroidism was slightly higher than the median incidence reported for conventional thyroidectomy (0% to 3%) (66). This could be related to the fact that the researchers in this study only used NIRAF imaging, which does not provide any information about the perfusion and viability of the glands.

All three cited studies were performed at high-volume expert centres, where permanent postoperative hypoparathyroidism is already relatively rare, so a larger sample size is necessary to capture statistically significant differences between the fluorescence imaging and control groups. Certain unaccounted for patient and tumour characteristics, such as age, BMI, size of the tumour and size and extent of nodal metastases, could have contributed to the fact that no difference in the frequency of permanent hypocalcemia was observed in the cited studies. More studies are needed to assess, if application of NIRAF imaging could reduce the rate of permanent hypocalcemia rate in T3-T4 tumours as compared to T1-T2. This would facilitate the decision on whether to proceed to a prophylactic CLND based on the tumour stage.

### ICG angiography and CLND

ICG angiography is used mainly to assess parathyroid perfusion and vitality. It has been shown that the presence of at least one well-perfused parathyroid gland is sufficient to prevent hypocalcaemia, and that the ICG-based scoring system has superiority over visual assessment (60). In the study by Ouyang et al, however, the negative predictive value for postoperative hypoparathyroidism in patients with at least one well-perfused gland was 89.7%, which is lower than in most other studies (63). This highlights the lack of standardisation of the ICG scale which relies on subjective visual evaluation. Further research is needed to develop an objective quantitative ICG scale. A valuable adjunct to the ICG scale in case of inconclusive results could be the fine-needle pricking test. Wu et al. studied a group of 608 patients in whom all parathyroid glands were identified during thyroidectomy with CLND (67). The presence of at least one well-vascularised parathyroid gland on fine-needle pricking test (FNP score 2) predicted normal postoperative parathyroid hormone concentrations, suggesting excellent predictive ability of the test. The group reported exceptionally positive outcomes: the frequency of transient hypoparathyroidism of 2% and no cases of permanent hypoparathyroidism, which suggests that thyroidectomy with CLND is a safe procedure when performed in expert centres, and that ICG angiography and/or the fine-needle pricking test are valuable adjuncts in decision-making on parathyroid gland autotranspantation.

A study recently published by Benmiloud et al. assessed the usefulness of ICG angiography in detecting the vascular pedicle of the parathyroid gland to guide dissection (68). An ICG bolus was administered after initial dissection and localisation of the parathyroid glands’ NIRAF. The aim was to identify the parathyroid blood-supplying vessel or the general pattern of vascular arborisation to guide further dissection. The obtained angiograms were than classified as iMAP 0, 1 and 2, with 2 meaning a clear view of the vascular pedicle flowing into the parathyroid gland. It has been shown that ICG provides clear information on the vascular supply (iMAP score 2) in about one-third of the cases. Further studies are needed to assess the optimal timepoint for the initial angiography: done too early, it will fail to reveal the vascular supply as it may still be covered by soft tissue. If ICG angiography is done too late, the vascular pedicle might already be damaged by dissection. In a study on 100 cadavers, Delattre et al. showed that the main parathyroid blood supply is at risk during standard parathyroidectomy in 38.2% of cases (69). In consequence, techniques of capsular dissection and preservation of the surrounding vasculature have been developed. Nevertheless, the ability to clearly identify the single feeding pedicle makes ICG angiography a valuable adjunct and allows to adjust the surgical dissection, which is of particular importance in interventions with a higher risk of hypoparathyroidism, such as cancer surgery with CLND.

### NIRAF and CLND

The results of the cited studies are in line with most published studies showing that NIRAF imaging helps lower the frequency of transient postoperative hypocalcaemia, but has no effect on the permanent hypocalcaemia (54–56, 70). The PARAFLUO multicentre randomized clinical trial with 241 patients who underwent thyroidectomy showed that NIRAF imaging reduced the incidence of transient hypocalcaemia from 21.7% to 9.1%, but had no effect on permanent hypocalcaemia (55). This could be due to the rarity of the event and the fact that the study was conducted at high-volume centres with already low rates of permanent hypoparathyroidism. Furthermore, the PARAFLUO trial showed that NIRAF imaging lowered the incidence of inadvertent parathyroidectomy from 11.7% to 2.5%, and the autotransplantation rate from 13.3% to 3.3%. A recent meta-analysis of eight studies and 2889 patients concluded that the use of NIRAF led to an almost three-fold decrease in the incidence of transient hypocalcemia, from 22.4% to 7.11%, and a nearly two-fold decrease in parathyroid gland resection rates, from 14.4% to 7.7%, with no impact on permanent hypocalcemia (71). Studies reporting positive effects of NIRAF imaging on the frequency of postoperative hypoparathyroidism are scarcer (58). This difference highlights the need for further studies, especially for evaluation of the usefulness of fluorescence imaging in thyroidectomy with lymph node dissection in low-volume centres. Another gap to address would be to compare the outcomes of NIRAF imaging in different surgical indication, like Graves’ disease versus thyroid cancer, as both of them are known to lead to relatively frequent hypoparathyroidism.

Fluorescence imaging can be used in many different ways during thyroidectomy with CLND (**Table 4**). First of all, NIRAF imaging can be used for early identification of the parathyroid glands, the so-called “mapping”. In one study, 46% of glands were invisible to the naked eye in the initial stages of the dissection because they were covered with soft tissue, but were identified with NIRAF imaging without any further dissection (51). Early mapping of parathyroid glands can guide dissection, allowing the surgeon to take special care not to damage or resect the parathyroid glands. In the special case of CLND, the parathyroid glands can be difficult to distinguish from lymph nodes with the naked eye. Adding NIRAF imaging to the naked-eye observation in patients undergoing total thyroidectomy was shown to improve the number of identified parathyroid glands from 2.5 to 3.7 per patient (52). The three studies reviewed here showed similar results, with more parathyroid glands identified with the help of fluorescence imaging (63–65). Additionally, NIRAF can be used to check for inadvertently resected glands in the specimen, as shown by Kim et al. and the PARAFLUO trial (55). Kim et al. claimed that NIRAF helped identify the inferior parathyroid glands early, so extra care was taken to preserve them *in situ (* 65). This is of particular importance in CLND where the inferior parathyroid glands might be difficult to distinguish from the lymph nodes. However, it is important to keep in mind that false positive results in fluorescence imaging have been described when lymph nodes were metastatic (72). Adding ICG angiography to this procedure might help identify the vascular pedicle supplying the inferior parathyroid gland, and provide precise guidance for the dissection. The preservation of the parathyroid gland vasculature might have a more important effect on the postoperative gland function than visual identification of the gland only.

**Table 4:** Summary of applications of NIRAF imaging and contrast-enhanced fluorescence angiography in thyroidectomy with CLND.

Imaging modality	Applications
**NIRAF imaging**	•To map the parathyroid glands early, and guide dissection in order to preserve the maximum number of the glands. •To distinguish the parathyroid glands from the lymph nodes. •To identify accidentally resected parathyroid glands in the specimen to enable autotransplantation.
**Contrast-enhanced fluorescence angiography**	•To identify the parathyroid gland blood supply early, so that dissection can be adapted to preserve it. •To assess the perfusion of the parathyroid glands after first lobectomy and ipsilateral CLND to determine the extension of resection on the contralateral side (if no parathyroid glands are preserved in the first lobectomy, prophylactic CLND on the contralateral side may be omitted). •To determine the parathyroid gland perfusion and the risk for hypoparathyroidism, at the end of the procedure. •To identify *in situ* devascularised parathyroid glands to support the decision on autotransplantation.

NIRAF, near-infrared fluorescence; CLND, central lymph node dissection. Adapted from *Solorzano et al.* (72).

## Conclusion

The use of fluorescence imaging in thyroidectomy with CLND for thyroid cancer facilitates the early identification of parathyroid glands, helps to identify more parathyroid glands per intervention, lowers the number of inadvertently resected glands and lowers the frequency of transient hypoparathyroidism. Further studies are needed to evaluate the technique’s impact on permanent hypoparathyroidism. A combination of different fluorescence imaging techniques (NIRAF to map parathyroid glands; ICG angiography to locate the vascular pedicle intraoperatively, and to evaluate gland perfusion postoperatively) might yield even better results. Fluorescence imaging can be useful to minimise the risk of hypoparathyroidism associated with CLND, while allowing to take advantage of all its potential benefits. With further development, fluorescence imaging techniques might increase the safety of thyroid cancer surgery with neck dissection and thus shift the paradigm to more frequently recommended prophylactic CLND.

## Author contributions

PK: Conceptualization (lead); writing – original draft (lead); formal analysis (lead); writing – review and editing (equal). MD, SL: writing – review and editing (equal). FT: Methodology (lead); writing – review and editing (equal). All authors contributed to the article and approved the submitted version.

## References

[1] Cancer today. Available at: http://gco.iarc.fr/today/home.

[2] Ahn HS Welch HG. South korea’s thyroid-cancer ‘Epidemic’–turning the tide. N Engl J Med (2015) 373:2389–90. doi: 10.1056/NEJMc1507622 26650173

[3] Kweon S-S Shin M-H Chung I-J Kim Y-J Choi J-S. Thyroid cancer is the most common cancer in women, based on the data from population-based cancer registries, south korea. jpn. J Clin Oncol (2013) 43:1039–46. doi: 10.1093/jjco/hyt102 23894204

[4] Lim H Devesa SS Sosa JA Check D Kitahara CM. Trends in thyroid cancer incidence and mortality in the united states, 1974-2013. JAMA (2017) 317:1338–48. doi: 10.1001/jama.2017.2719 PMC8216772 28362912

[5] Haugen BR Alexander EK Bible KC Doherty GM Mandel SJ Nikiforov YE. 2015 American Thyroid association management guidelines for adult patients with thyroid nodules and differentiated thyroid cancer: The American thyroid association guidelines task force on thyroid nodules and differentiated thyroid cancer. Thyroid (2016) 26:1–133. doi: 10.1089/thy.2015.0020 26462967 PMC4739132

[6] Ito Y Jikuzono T Higashiyama T Asahi S Tomoda C Takamura Y. Clinical significance of lymph node metastasis of thyroid papillary carcinoma located in one lobe. World J Surg (2006) 30:1821–8. doi: 10.1007/s00268-006-0211-5 16983469

[7] Liu Y Wang Y Zhao K Li D Chen Z Jiang R. Lymph node metastasis in young and middle-aged papillary thyroid carcinoma patients: a SEER-based cohort study. BMC Cancer (2020) 20:181. doi: 10.1186/s12885-020-6675-0 32131769 PMC7057480

[8] Roh J-L Kim J-M Park CI. Central cervical nodal metastasis from papillary thyroid microcarcinoma: Pattern and factors predictive of nodal metastasis. Ann Surg Oncol (2008) 15:2482–6. doi: 10.1245/s10434-008-0044-6 18612697

[9] Wada N Duh QY Sugino K Iwasaki H Kameyama K Mimura T. Lymph node metastasis from 259 papillary thyroid microcarcinomas: frequency, pattern of occurrence and recurrence, and optimal strategy for neck dissection. Ann Surg (2003) 237:399–407. doi: 10.1097/01.SLA.0000055273.58908.19 12616125 PMC1514312

[10] Barney BM Hitchcock YJ Sharma P Shrieve DC Tward JD. Overall and cause-specific survival for patients undergoing lobectomy, near-total, or total thyroidectomy for differentiated thyroid cancer. Head Neck (2011) 33:645–9. doi: 10.1002/hed.21504 20687168

[11] Zaydfudim V Feurer ID Griffin MR Phay JE. The impact of lymph node involvement on survival in patients with papillary and follicular thyroid carcinoma. Surgery (2008) 144:1070–1077; discussion 1077-1078. doi: 10.1016/j.surg.2008.08.034 19041020

[12] Podnos YD Smith D Wagman LD Ellenhorn JDI. The implication of lymph node metastasis on survival in patients with well-differentiated thyroid cancer. Am Surg (2005) 71:731–4. doi: 10.1177/000313480507100907 16468507

[13] Lundgren CI Hall P Dickman PW Zedenius J. Clinically significant prognostic factors for differentiated thyroid carcinoma: A population-based, nested case-control study. Cancer (2006) 106:524–31. doi: 10.1002/cncr.21653 16369995

[14] Bozec A Dassonville O Chamorey E Poissonnet G Sudaka A Peyrottes I. Clinical impact of cervical lymph node involvement and central neck dissection in patients with papillary thyroid carcinoma: a retrospective analysis of 368 cases. Eur Arch Oto-Rhino-Laryngol. Off J Eur Fed. Oto-Rhino-Laryngol. Soc EUFOS Affil. Ger. Soc Oto-Rhino-Laryngol. - Head Neck Surg (2011) 268:1205–12. 10.1007/s00405-011-1639-2 21607578

[15] Adam MA Pura J Goffredo P Dinan MA Reed SD Scheri RP. Presence and number of lymph node metastases are associated with compromised survival for patients younger than age 45 years with papillary thyroid cancer. J Clin Oncol Off J Am Soc Clin Oncol (2015) 33:2370–5. doi: 10.1200/JCO.2014.59.8391 26077238

[16] Hughes CJ Shaha AR Shah JP Loree TR. Impact of lymph node metastasis in differentiated carcinoma of the thyroid: a matched-pair analysis. Head Neck (1996) 18:127–32. doi: 10.1002/(SICI)1097-0347(199603/04)18:2<127::AID-HED3>3.0.CO;2-3 8647677

[17] Schneider DF Elfenbein D Lloyd RV Chen H Sippel RS. Lymph node metastases do not impact survival in follicular variant papillary thyroid cancer. Ann Surg Oncol (2015) 22:158–63. doi: 10.1245/s10434-014-3964-3 PMC4275358 25092163

[18] Ito Y Tomoda C Uruno T Takamura Y Miya A Kobayashi K. Clinical significance of metastasis to the central compartment from papillary microcarcinoma of the thyroid. World J Surg (2006) 30:91–9. doi: 10.1007/s00268-005-0113-y 16369721

[19] Lee YC Na SY Park GC Han JH Kim SW Eun YG. Occult lymph node metastasis and risk of regional recurrence in papillary thyroid cancer after bilateral prophylactic central neck dissection: A multi-institutional study. Surgery (2017) 161:465–71. doi: 10.1016/j.surg.2016.07.031 27574773

[20] Sywak M Cornford L Roach P Stalberg P Sidhu S Delbridge L. Routine ipsilateral level VI lymphadenectomy reduces postoperative thyroglobulin levels in papillary thyroid cancer. Surgery (2006) 140:1000–1005; discussion 1005-1007. doi: 10.1016/j.surg.2006.08.001 17188149

[21] Pacini F Schlumberger M Dralle H Elisei R Smit JW Wiersinga W. European Consensus for the management of patients with differentiated thyroid carcinoma of the follicular epithelium. Eur J Endocrinol (2006) 154:787–803. doi: 10.1530/eje.1.02158 16728537

[22] Cooper DS Doherty GM Haugen BR Kloos RT Lee SL Mandel SJ American Thyroid Association (ATA) Guidelines Taskforce on Thyroid Nodules and Differentiated Thyroid Cancer Cooper DS Doherty GM Haugen BR Kloos RT Lee SL. Revised American thyroid association management guidelines for patients with thyroid nodules and differentiated thyroid cancer. Thyroid Off J Am Thyroid Assoc (2009) 19:1167–214. doi: 10.1089/thy.2009.0110 19860577

[23] Hartl DM Al Ghuzlan A Bidault S Breuskin I Guerlain J Girard E. Risk staging with prophylactic unilateral central neck dissection in low-risk papillary thyroid carcinoma. Eur J Surg Oncol J Eur Soc Surg Oncol Br Assoc Surg Oncol (2023) 49(3):568–74. doi: 10.1016/j.ejso.2022.11.007 36411174

[24] Leboulleux S Bournaud C Chougnet CN Zerdoud S Al Ghuzlan A Catargi B. Thyroidectomy without radioiodine in patients with low-risk thyroid cancer. N Engl J Med (2022) 386:923–32. doi: 10.1056/NEJMoa2111953 35263518

[25] Carling T Carty SE Ciarleglio MM Cooper DS Doherty GM Kim LT. American Thyroid association design and feasibility of a prospective randomized controlled trial of prophylactic central lymph node dissection for papillary thyroid carcinoma. Thyroid Off J Am Thyroid Assoc (2012) 22:237–44. doi: 10.1089/thy.2011.0317 22313454

[26] Sancho JJ Lennard TWJ Paunovic I Triponez F Sitges-Serra A. Prophylactic central neck disection in papillary thyroid cancer: A consensus report of the European society of endocrine surgeons (ESES). Langenbecks Arch Surg (2014) 399:155–63. doi: 10.1007/s00423-013-1152-8 24352594

[27] Zhao W You L Hou X Chen S Ren X Chen G. The effect of prophylactic central neck dissection on locoregional recurrence in papillary thyroid cancer after total thyroidectomy: A systematic review and meta-Analysis: pCND for the locoregional recurrence of papillary thyroid cancer. Ann Surg Oncol (2017) 24:2189–98. doi: 10.1245/s10434-016-5691-4 27913945

[28] Lang BH Ng SH Lau LL Cowling BJ Wong KP Wan KY. A systematic review and meta-analysis of prophylactic central neck dissection on short-term locoregional recurrence in papillary thyroid carcinoma after total thyroidectomy. Thyroid Off J Am Thyroid Assoc (2013) 23:1087–98. doi: 10.1089/thy.2012.0608 23402640

[29] Barczyński M Konturek A Stopa M Nowak W. Prophylactic central neck dissection for papillary thyroid cancer. Br J Surg (2013) 100:410–8. doi: 10.1002/bjs.8985 23188784

[30] Popadich A Levin O Lee JC Smooke-Praw S Ro K Fazel M. A multicenter cohort study of total thyroidectomy and routine central lymph node dissection for cN0 papillary thyroid cancer. Surgery (2011) 150:1048–57. doi: 10.1016/j.surg.2011.09.003 22136820

[31] Hartl DM Mamelle E Borget I Leboulleux S Mirghani H Schlumberger M. Influence of prophylactic neck dissection on rate of retreatment for papillary thyroid carcinoma. World J Surg (2013) 37:1951–8. doi: 10.1007/s00268-013-2089-3 23677562

[32] Nixon IJ Ganly I Patel SG Morris LG Palmer FL Thomas D. Observation of clinically negative central compartment lymph nodes in papillary thyroid carcinoma. Surgery (2013) 154:1166–1172; discussion 1172-1173. doi: 10.1016/j.surg.2013.04.035 24238042

[33] Zetoune T Keutgen X Buitrago D Aldailami H Shao H Mazumdar M. Prophylactic central neck dissection and local recurrence in papillary thyroid cancer: a meta-analysis. Ann Surg Oncol (2010) 17:3287–93. doi: 10.1245/s10434-010-1137-6 20596784

[34] Yang P Li J Jing H Chen Q Song X Qian L. Effect of prophylactic central lymph node dissection on locoregional recurrence in patients with papillary thyroid microcarcinoma. Int J Endocrinol (2021) 2021:8270622. doi: 10.1155/2021/8270622 34819955 PMC8608519

[35] Viola D Materazzi G Valerio L Molinaro E Agate L Faviana P. Prophylactic central compartment lymph node dissection in papillary thyroid carcinoma: clinical implications derived from the first prospective randomized controlled single institution study. J Clin Endocrinol Metab (2015) 100:1316–24. doi: 10.1210/jc.2014-3825 25590215

[36] Sanabria A Betancourt-Agüero C Sánchez-Delgado JG García-Lozano C. Prophylactic central neck lymph node dissection in low-risk thyroid carcinoma patients does not decrease the incidence of locoregional recurrence: A meta-analysis of randomized trials. Ann Surg (2022) 276:66–73. doi: 10.1097/SLA.0000000000005388 35129470

[37] Nixon IJ Ganly I Palmer FL Whitcher MM Patel SG Tuttle RM. Disease-related death in patients who were considered free of macroscopic disease after initial treatment of well-differentiated thyroid carcinoma. Thyroid Off J Am Thyroid Assoc (2011) 21:501–4. doi: 10.1089/thy.2010.0451 21476889

[38] Dobrinja C Troian M Cipolat Mis T Rebez G Bernardi S Fabris B. Rationality in prophylactic central neck dissection in clinically node-negative (cN0) papillary thyroid carcinoma: Is there anything more to say? a decade experience in a single-center. Int J Surg Lond Engl (2017) 41 Suppl 1:S40–7. doi: 10.1016/j.ijsu.2017.01.113 28506412

[39] Hall CM Snyder SK Maldonado YM Lairmore TC. Routine central lymph node dissection with total thyroidectomy for papillary thyroid cancer potentially minimizes level VI recurrence. Surgery (2016) 160:1049–58. doi: 10.1016/j.surg.2016.06.042 27521047

[40] Ahn J-H Kwak JH Yoon SG Yi JW Yu HW Kwon H. A prospective randomized controlled trial to assess the efficacy and safety of prophylactic central compartment lymph node dissection in papillary thyroid carcinoma. Surgery (2022) 171:182–9. doi: 10.1016/j.surg.2021.03.071 34391573

[41] Salem FA Bergenfelz A Nordenström E Almquist M. Central lymph node dissection and permanent hypoparathyroidism after total thyroidectomy for papillary thyroid cancer: population-based study. Br J Surg (2021) 108(6):684–90. doi: 10.1002/bjs.12028 34157088

[42] Hadedeya D Kay J Attia A Omar M Shalaby M Youssef MR. Effect of postsurgical chronic hypoparathyroidism on morbidity and mortality: a systematic review and meta-analysis. Gland Surg (2021) 10:3007–19. doi: 10.21037/gs-21-181 PMC8575699 34804887

[43] Bilezikian JP. Hypoparathyroidism. J Clin Endocrinol Metab (2020) 105(6):dgaa113. doi: 10.1210/clinem/dgaa113 32322899 PMC7176479

[44] Almquist M Ivarsson K Nordenström E Bergenfelz A. Mortality in patients with permanent hypoparathyroidism after total thyroidectomy. Br J Surg (2018) 105(10):1313–8. doi: 10.1002/bjs.10843 29663312

[45] Bergenfelz A Nordenström E Almquist M. Morbidity in patients with permanent hypoparathyroidism after total thyroidectomy. Surgery (2020) 167(1):124–8. doi: 10.1016/j.surg.2019.06.056 31570150

[46] Fanget F Demarchi MS Maillard L El Boukili I Gerard M Decaussin M. Hypoparathyroidism: Consequences, economic impact, and perspectives. a case series and systematic review. Ann Endocrinol (2021) 82:572–81. doi: 10.1016/j.ando.2021.07.085 34400129

[47] Paras C Keller M White L Phay J Mahadevan-Jansen A. Near-infrared autofluorescence for the detection of parathyroid glands. J Biomed Opt (2011) 16:067012. doi: 10.1117/1.3583571 21721833

[48] McWade MA Paras C White LM Phay JE Mahadevan-Jansen A Broome JT. A novel optical approach to intraoperative detection of parathyroid glands. Surgery (2013) 154(6):1371–7. doi: 10.1016/j.surg.2013.06.046 PMC3898879 24238054

[49] McWade MA Paras C White LM Phay JE Solórzano CC Broome JT. Label-free intraoperative parathyroid localization with near-infrared autofluorescence imaging. J Clin Endocrinol Metab (2014) 99:4574–80. doi: 10.1210/jc.2014-2503 PMC4255111 25148235

[50] McWade MA Sanders ME Broome JT Solórzano CC Mahadevan-Jansen A. Establishing the clinical utility of autofluorescence spectroscopy for parathyroid detection. Surgery (2016) 159:193–202. doi: 10.1016/j.surg.2015.06.047 26454675 PMC4836056

[51] Kahramangil B Dip F Benmiloud F Falco J de La Fuente M Verna S. Detection of parathyroid autofluorescence using near-infrared imaging: A multicenter analysis of concordance between different surgeons. Ann Surg Oncol (2018) 25:957–62. doi: 10.1245/s10434-018-6364-2 29411199

[52] Falco J Dip F Quadri P de la Fuente M Prunello M Rosenthal RJ. Increased identification of parathyroid glands using near infrared light during thyroid and parathyroid surgery. Surg Endosc (2017) 31:3737–42. doi: 10.1007/s00464-017-5424-1 28364157

[53] Spare parathyroid glands during thyroid surgery with perioperative autofluorescence imaging: A diagnostic study. 10.1007/s00268-021-06102-7 33835219

[54] Benmiloud F Rebaudet S Varoquaux A Penaranda G Bannier M Denizot A. Impact of autofluorescence-based identification of parathyroids during total thyroidectomy on postoperative hypocalcemia: a before and after controlled study. Surgery (2018) 163:23–30. doi: 10.1016/j.surg.2017.06.022 29122325

[55] Benmiloud F Godiris-Petit G Gras R Gillot JC Turrin N Penaranda G. Association of autofluorescence-based detection of the parathyroid glands during total thyroidectomy with postoperative hypocalcemia risk: Results of the PARAFLUO multicenter randomized clinical trial. JAMA Surg (2020) 155:106–12. doi: 10.1001/jamasurg.2019.4613 PMC6865247 31693081

[56] Dip F Falco J Verna S Prunello M Loccisano M Quadri P. Randomized controlled trial comparing white light with near-infrared autofluorescence for parathyroid gland identification during total thyroidectomy. J Am Coll Surg (2019) 228:744–51. doi: 10.1016/j.jamcollsurg.2018.12.044 30710614

[57] Kim YS Erten O Kahramangil B Aydin H Donmez M Berber E. The impact of near infrared fluorescence imaging on parathyroid function after total thyroidectomy. J Surg Oncol (2020) 122:973–9. doi: 10.1002/jso.26098 32602151

[58] Papavramidis TS Chorti A Tzikos G Anagnostis P Pantelidis P Pliakos I. The effect of intraoperative autofluorescence monitoring on unintentional parathyroid gland excision rates and postoperative PTH concentrations-a single-blind randomized-controlled trial. Endocrine (2021) 72:546–52. doi: 10.1007/s12020-020-02599-5 33432503

[59] Wolf HW Runkel N Limberger K Nebiker CA. Near-infrared autofluorescence of the parathyroid glands during thyroidectomy for the prevention of hypoparathyroidism: a prospective randomized clinical trial. Langenbecks Arch Surg (2022) 407:3031–8. doi: 10.1007/s00423-022-02624-3 PMC9640439 35904639

[60] Vidal Fortuny J Belfontali V Sadowski SM Karenovics W Guigard S Triponez F. Parathyroid gland angiography with indocyanine green fluorescence to predict parathyroid function after thyroid surgery. Br J Surg (2016) 103:537–43. doi: 10.1002/bjs.10101 PMC5067567 26864909

[61] Vidal Fortuny J Sadowski SM Belfontali V Guigard S Poncet A Ris F. Randomized clinical trial of intraoperative parathyroid gland angiography with indocyanine green fluorescence predicting parathyroid function after thyroid surgery. Br J Surg (2018) 105:350–7. doi: 10.1002/bjs.10783 PMC6084300 29405252

[62] Rudin AV McKenzie TJ Thompson GB Farley DR Lyden ML. Evaluation of parathyroid glands with indocyanine green fluorescence angiography after thyroidectomy. World J Surg (2019) 43:1538–43. doi: 10.1007/s00268-019-04909-z 30659346

[63] Ouyang H Wang B Sun B Cong R Xia F Li X. Application of indocyanine green angiography in bilateral axillo-breast approach robotic thyroidectomy for papillary thyroid cancer. Front Endocrinol (2022) 13:916557. doi: 10.3389/fendo.2022.916557 PMC9260684 35813620

[64] Yin S Pan B Yang Z Tang M Mo H Li Y. Combined use of autofluorescence and indocyanine green fluorescence imaging in the identification and evaluation of parathyroid glands during total thyroidectomy: A randomized controlled trial. Front Endocrinol (2022) 13:897797. doi: 10.3389/fendo.2022.897797 PMC9243533 35784544

[65] Kim DH Kim SW Kang P Choi J Lee HS Park SY. Near-infrared autofluorescence imaging may reduce temporary hypoparathyroidism in patients undergoing total thyroidectomy and central neck dissection. Thyroid Off J Am Thyroid Assoc (2021) 31:1400–8. doi: 10.1089/thy.2021.0056 33906431

[66] Edafe O Antakia R Laskar N Uttley L Balasubramanian SP. Systematic review and meta-analysis of predictors of post-thyroidectomy hypocalcaemia. Br J Surg (2014) 101:307–20. doi: 10.1002/bjs.9384 24402815

[67] Wu Y-J Wang JB Li FB Jin L Zhou L Xie L. Fine-needle pricking test of the parathyroid gland during thyroid surgery in predicting parathyroid function. Int J Endocrinol (2022) 2022:8747680. doi: 10.1155/2022/8747680 35795846 PMC9252692

[68] Benmiloud F Penaranda G Chiche L Rebaudet S. Intraoperative mapping angiograms of the parathyroid glands using indocyanine green during thyroid surgery: Results of the fluogreen study. World J Surg (2022) 46:416–24. doi: 10.1007/s00268-021-06353-4 34743241

[69] Delattre JF Flament JB Palot JP Pluot M. [Variations in the parathyroid glands. number, situation and arterial vascularization. anatomical study and surgical application]. J Chir. (Paris) (1982) 119:633–41. 7153263

[70] Ladurner R Al Arabi N Guendogar U Hallfeldt K Stepp H Gallwas J. Near-infrared autofluorescence imaging to detect parathyroid glands in thyroid surgery. Ann R Coll Surg Engl (2018) 100:33–6. doi: 10.1308/rcsann.2017.0102 PMC5838663 29022781

[71] Lu W Chen Q Zhang P Su A Zhu J. Near-infrared autofluorescence imaging in thyroid surgery: A systematic review and meta-analysis. J Investig Surg Off J Acad Surg Res (2022) 35:1723–32. doi: 10.1080/08941939.2022.2095468 35786292

[72] Solórzano CC Thomas G Berber E Wang TS Randolph GW Duh QY. Current state of intraoperative use of near infrared fluorescence for parathyroid identification and preservation. Surgery (2021) 169:868–78. doi: 10.1016/j.surg.2020.09.014 PMC7987670 33139065

